# Supplementary material for: Vaccinomics-Aided Development of a Next-Generation Chimeric Vaccine against an Emerging Threat: Mycoplasma genitalium
Source: Vaccines (Basel). 2022 Oct 14;10(10):1720. doi: 10.3390/vaccines10101720 (PMC9608589; doi:10.3390/vaccines10101720)
Supplement: Supplementary file 1 [file vaccines-10-01720-s001.zip › vaccines-1942977-supplementary.pdf]

Supplementary Data

# Vaccinomics-Aided Development of a Next-Generation Chimeric Vaccine against an Emerging Threat: *Mycoplasma genitalium*

Table S1. Selected *Mycoplasma genitalium* proteins

| **Sr. No** | **Accession no.** | **Protein** | | **Amino acid** | **VaxiJen score** | **Remarks** | **Cell2Go** |
|---|---|---|---|---|---|---|---|
| **1** | P47377 | Uncharacterized protein MG131 | MG131 | 74 | 1.1648 | Probable ANTIGEN | Plasma membrane |
| **2** | P58061 | Probable protein-export membrane protein SecG | SECG | 77 | 0.9311 | Probable ANTIGEN | Inner membrane/ Plasmamembrane |
| **3** | P47603 | RL32_MYCGE 50S ribosomal protein L32 | | 57 aa | 1.1024 | Probable ANTIGEN | Inner membrane/ cytoplasmic |
| **3** | P47489 | Glycerol-3-phosphate acyltransferase | PLSY | | 0.9202 | Probable ANTIGEN | plasma membrane |
| **4** | UNIPROT ID-Q9ZB71 | Uncharacterized protein MG384.1 | Y384A | 149 | 1.0589 | Probable ANTIGEN | plasma membrane |
| **5** | P47629 | Hypothetical protein 1 | HP1 | | | | outer membrane |
| **6** | WP_009885924.1 | Hypothetical protein 2 | HP2 | | | | outer membrane |
| **7** | WP_014894048.1 | Hypothetical protein 3 | HP3 | | | | outer membrane |
| **8** | P20796 | MGPa | | | | | |


| **Sr. No** | **Accession no.** | **Protein** | | **Amino acid** | **VaxiJen score** | **Remarks** | **Cell2Go** |
|---|---|---|---|---|---|---|---|
| **1** | P47377 | Uncharacterized protein MG131 | MG131 | 74 | 1.1648 | Probable ANTIGEN | Plasma membrane |
| **2** | P58061 | Probable protein-export membrane protein SecG | SECG | 77 | 0.9311 | Probable ANTIGEN | Inner membrane/ Plasmamembrane |
| **3** | P47603 | RL32_MYCGE 50S ribosomal protein L32 | | 57 aa | 1.1024 | Probable ANTIGEN | Inner membrane/ cytoplasmic |
| **3** | P47489 | Glycerol-3-phosphate acyltransferase | PLSY | | 0.9202 | Probable ANTIGEN | plasma membrane |
| **4** | UNIPROT ID-Q9ZB71 | Uncharacterized protein MG384.1 | Y384A | 149 | 1.0589 | Probable ANTIGEN | plasma membrane |
| **5** | P47629 | Hypothetical protein 1 | HP1 | | | | outer membrane |
| **6** | WP_009885924.1 | Hypothetical protein 2 | HP2 | | | | outer membrane |
| **7** | WP_014894048.1 | Hypothetical protein 3 | HP3 | | | | outer membrane |
| **8** | P20796 | MGPa | | | | | |


Table S2. CTL (9-mer) and HTLs selection

Supertype A1

| Sr.No | Protein ID | Peptide | Binding affinity | Affinity rescale | Cleavage | Tap transporter efficiency | Combined score |
|---|---|---|---|---|---|---|---|
| 1 | sp_P47377_Y1 | YSALIPLFI | 0.2467 | 1.0475 | 0.0755 | 0.5230 | 1.0849 |
| 2 | | IVKILFFAY | 0.1147 | 0.4871 | 0.9700 | 3.0350 | 0.7843 |
| 3 | | CIDFLALIL | 0.1769 | 0.7509 | 0.3413 | 0.3413 | 0.8140 |
| 4 | sp_P47489_PL | YQSTYFLSY | 0.5992 | 2.5439 | 0.9740 | 3.0230 | 2.8412 |
| 5 | | FAATIGHIF | 0.1518 | 0.6444 | 0.1412 | 2.5020 | 0.7907 |
| 6 | | TIGHIFPLY | 0.1626 | 0.6902 | 0.9419 | 3.0230 | 0.9826 |
| 7 | | IMITLITKY | 0.1542 | 0.6545 | 0.9254 | 3.0820 | 0.9474 |
| 8 | | IILIPWLDY | 0.1127 | 0.4783 | 0.9630 | 3.1410 | 0.7798 |
| 9 | | LIPWLDYLY | 0.1867 | 0.7926 | 0.9731 | 3.0510 | 1.0911 |
| 10 | | SITYQNEWY | 0.2670 | 1.1336 | 0.0712 | 3.1710 | 1.3029 |
| 11 | sp_Q9ZB71_Y3 | FLFVPLLIY | 0.1681 | 0.7139 | 0.8914 | 2.9050 | 0.9928 |
| 12 | | FTKYIIWEL | 0.1503 | 0.6382 | 0.6651 | 0.9900 | 0.7875 |
| 13 | | YSTAFTLIV | 0.3090 | 1.3120 | 0.8728 | 0.2920 | 1.4575 |
| **A2 superfamily** | | | | | | | |
| 14 | sp_P47377_Y1 | MQYSALIPL | 0.7405 | 1.1038 | 0.8706 | 1.2620 | 1.2975 |
| 15 | | ALIPLFILL | 0.7399 | 1.1029 | 0.7368 | 1.2410 | 1.2755 |
| 16 | | LIPLFILLI | 0.4078 | 0.6079 | 0.8216 | 0.8060 | 0.7715 |
| 17 | | FILLISLVL | 0.6209 | 0.9255 | 0.9174 | 1.0540 | 1.1158 |
| 18 | | QIVKILFFA | 0.6010 | 0.8959 | 0.0473 | -0.1750 | 0.8943 |
| 19 | | KILFFAYCI | 0.5971 | 0.8900 | 0.4294 | 0.8350 | 0.9962 |
| 20 | | FFAYCIDFL | 0.5000 | 0.7454 | 0.0680 | 1.0920 | 0.8102 |
| 21 | | FAYCIDFLA | 0.6208 | 0.9254 | 0.0529 | -0.4960 | 0.9085 |
| 22 | | YCIDFLALI | 0.5045 | 0.7521 | 0.1618 | 0.7580 | 0.8142 |
| 23 | | FLALILAVM | 0.6286 | 0.9370 | 0.9049 | 0.1070 | 1.078 |
| 24 | | ALILAVMLL | 0.5756 | 0.8580 | 0.9477 | 1.2350 | 1.0620 |

| | | | | | | | |
|---|---|---|---|---|---|---|---|
| 25 | | LLTFLSHGL | 0.5316 | 0.7924 | 0.9514 | 0.9780 | 0.9840 |
| 26 | | FLSHGLLSL | 0.8486 | 1.2650 | 0.9146 | 0.8850 | 1.4464 |
| 27 | | GLLSLAILI | 0.6375 | 0.9503 | 0.0806 | 0.3700 | 0.9809 |
| 28 | | LSLAILIPV | 0.6218 | 0.9269 | 0.5198 | 0.4250 | 1.0261 |
| 29 | | SLAILIPVL | 0.6520 | 0.9719 | 0.9643 | 1.0920 | 1.1712 |
| | sp_P58061_SEpep | QIVMFIMAV | 0.5613 | 0.8368 | 0.2926 | 0.5430 | 0.9078 |
| | | IVMFIMAVI | 0.4867 | 0.7256 | 0.2710 | 0.7120 | 0.8018 |
| | | FIMAVICLI | 0.8114 | 1.2096 | 0.2202 | 0.6690 | 1.2761 |
| | | IMAVICLII | 0.6118 | 0.9119 | 0.3633 | 0.5910 | 0.9960 |
| | | AVICLIIGL | 0.5726 | 0.8535 | 0.8852 | 1.3420 | 1.0534 |
| | | SLSGQDLEI | 0.5492 | 0.8186 | 0.9208 | 0.7570 | 0.9946 |
| | | KILQIIMFI | 0.7869 | 1.1731 | 0.2545 | 0.8350 | 1.2530 |
| | | ILQIIMFIL | 0.6250 | 0.9317 | 0.2687 | 1.0660 | 1.0253 |
| | | LQIIMFILV | 0.5415 | 0.8072 | 0.2709 | 0.4720 | 0.8714 |
| | | QIIMFILVV | 0.5934 | 0.8846 | 0.3668 | 0.5730 | 0.968 |
| | | IIMFILVVL | 0.5781 | 0.8618 | 0.8060 | 1.1220 | 1.0388 |
| | | FILVVLFLI | 0.7618 | 1.1356 | 0.2226 | 0.6220 | 1.2001 |
| | | ILVVLFLIL | 0.4340 | 0.6469 | 0.8151 | 0.9700 | 0.8177 |
| | | VVLFLILGL | 0.5267 | 0.7852 | 0.7392 | 1.2210 | 0.9571 |
| | | VLFLILGLI | 0.4548 | 0.6780 | 0.7093 | 0.7750 | 0.8231 |
| | sp_P47603_RL | ALTLQTLSV | 0.6564 | 0.9785 | 0.9647 | 0.3030 | 1.138 |
| | sp_P47489_PL | IAILVIFSL | 0.4405 | 0.6567 | 0.9692 | 1.2530 | 0.8647 |
| | | FSLASGYLL | 0.4650 | 0.6932 | 0.2849 | 0.9270 | 0.7823 |
| | | YLLGSIIFA | 0.8782 | 1.3091 | 0.3212 | 0.5480 | 1.3299 |
| | | FAFLLTWIL | 0.5558 | 0.8285 | 0.9138 | 0.9190 | 1.011 |
| | | FLLTWILFR | 0.5098 | 0.7599 | 0.4207 | 1.5810 | 0.9021 |
| | | LLTWILFRF | 0.3495 | 0.5210 | 0.9520 | 2.4170 | 0.7846 |

| | | | | | | | |
|---|---|---|---|---|---|---|---|
| | | KVYQSTYFL | 0.7294 | 1.0873 | 0.9548 | 1.2200 | 1.2915 |
| | | FLSYLSCFA | 0.7180 | 1.0703 | 0.0725 | 0.6670 | 1.0479 |
| | | YLSCFAATI | 0.7319 | 1.0910 | 0.4521 | 0.5840 | 1.1880 |
| | | ATIGHIFPL | 0.6785 | 1.0115 | 0.9616 | 1.3000 | 1.2207 |
| | | AISLWWFLI | 0.5115 | 0.7625 | 0.4800 | 0.8510 | 0.8771 |
| | | SLWWFLICL | 0.8144 | 1.2140 | 0.9523 | 1.2080 | 1.4172 |
| | | FLICLLIWI | 0.8402 | 1.2525 | 0.0966 | 0.4720 | 1.2906 |
| | | LLIWIMITL | 0.7827 | 1.1668 | 0.9374 | 1.0400 | 1.3594 |
| | | LIWIMITLI | 0.5380 | 0.8021 | 0.4180 | 0.8870 | 0.9091 |
| | | MITLITKYV | 0.4938 | 0.7360 | 0.8925 | 0.4630 | 0.8931 |
| | | TLITKYVSL | 0.5770 | 0.8601 | 0.8838 | 1.0310 | 1.0442 |
| | | LASLITFFV | 0.6288 | 0.9374 | 0.5768 | 0.4210 | 1.0450 |
| | | SLITFFVLA | 0.6459 | 0.9628 | 0.4173 | -0.2400 | 1.0134 |
| | | LITFFVLAV | 0.5968 | 0.8896 | 0.2390 | 0.4650 | 0.948 |
| | | FVLAVIILI | 0.7483 | 1.1154 | 0.1202 | 0.4910 | 1.158 |
| | | AVIILIPWL | 0.4962 | 0.7396 | 0.3127 | 1.3540 | 0.8542 |
| | | ILIPWLDYL | 0.8419 | 1.2550 | 0.9129 | 1.0750 | 1.4456 |
| | | YQNEWYIIL | 0.7139 | 1.0641 | 0.9307 | 0.9260 | 1.2500 |
| | | CLWYWPLT | 0.7378 | 1.0998 | 0.6617 | 0.3600 | 1.217 |
| | | | | | | | |
| | sp_Q9ZB71_Y3 | LVLLWFLFV | 0.6507 | 0.9700 | 0.1552 | 0.3360 | 1.0101 |
| | | LLWFLFVPL | 0.7032 | 1.0482 | 0.9636 | 1.1970 | 1.2526 |
| | | YLFLAFFLF | 0.4275 | 0.6373 | 0.8934 | 2.6550 | 0.9040 |
| | | FLAFFLFAF | 0.4713 | 0.7026 | 0.3683 | 2.5240 | 0.8840 |
| | | FLFAFTKYI | 0.8591 | 1.2807 | 0.5535 | 0.4660 | 1.3870 |
| | | FTKYIIWEL | 0.6472 | 0.9647 | 0.6651 | 0.9900 | 1.1140 |
| | | YSTAFTLIV | 0.4952 | 0.7382 | 0.8728 | 0.2920 | 0.8837 |
| | | FLSGIIPMA | 0.8747 | 1.3038 | 0.4675 | -0.5670 | 1.3456 |

| | | | | | | | |
|---|---|---|---|---|---|---|---|
| | | IILLRFFLV | 0.6244 | 0.930<br>7 | 0.6475 | 0.4280 | 1.0493 |
| | | ILLRFFLV<br>L | 0.6262 | 0.933<br>5 | 0.8877 | 1.0240 | 1.1178 |
| | | FLVLITLM<br>L | 0.7600 | 1.132<br>8 | 0.9494 | 0.8520 | 1.3179 |
| | | FLVLITLM<br>L | 0.7600 | 1.132<br>8 | 0.9494 | 0.8520 | 1.3179 |
| | | ITLMLSFF<br>L | 0.5452 | 0.812<br>7 | 0.1962 | 1.0220 | 0.8933 |
| | | TLMLSFFL<br>L | 0.7213 | 1.075<br>3 | 0.8360 | 1.0270 | 1.2520 |
| | | FLIRSKYA<br>I | 0.6680 | 0.995<br>7 | 0.1326 | 0.4230 | 1.0368 |
| | | KLQQIQSD<br>L | 0.3987 | 0.594<br>4 | 0.9739 | 1.0000 | 0.7905 |
| | | KSAEIVFK<br>I | 0.4445 | 0.662<br>5 | 0.9356 | 0.8000 | 0.8429 |
| | | | | | | | |
| **A3 superfamily** | | | | | | | |
| | P47377_Y1 | LVLFCFSF<br>R | 0.6076 | 1.143<br>5 | 0.2715 | 1.6990 | 1.2692 |
| | | VLFCFSFR<br>K | 0.8276 | 1.557<br>6 | 0.9761 | 0.7060 | 1.7393 |
| | | IVKILFFA<br>Y | 0.3858 | 0.726<br>0 | 0.9700 | 3.0350 | 1.0233 |
| | | VMLLTFL<br>SH | 0.4286 | 0.806<br>6 | 0.3607 | -0.1800 | 0.8517 |
| | P58061_SE | KTKDRGF<br>VK | 0.5843 | 1.099<br>7 | 0.5543 | 0.6800 | 1.2168 |
| | | GLIFSFAP<br>R | 0.5136 | 0.966<br>5 | 0.8890 | 1.4420 | 1.1720 |
| | P47603_RL | AVQQRRS<br>SK | 0.5306 | 0.998<br>6 | 0.8453 | 0.7510 | 1.1630 |
| | | RSSKHRR<br>D | 0.5443 | 1.024<br>4 | 0.8214 | 0.8770 | 1.1915 |
| | | TLQTLSVC<br>K | 0.5423 | 1.020<br>7 | 0.8953 | 0.4790 | 1.1789 |
| | | SVCKKCG<br>K | 0.3662 | 0.689<br>2 | 0.9188 | 0.7130 | 0.8627 |
| | | GMYGELR<br>V | 0.6853 | 1.289<br>8 | 0.8972 | 0.5870 | 1.4537 |
| | P47489_PL | VIFSLASG<br>Y | 0.4705 | 0.885<br>6 | 0.8596 | 3.2750 | 1.1783 |
| | | IIFADIFSK | 0.7929 | 1.492<br>4 | 0.6993 | 0.7820 | 1.6363 |
| | | DIFSKILK<br>K | 0.5496 | 1.034<br>4 | 0.9544 | 0.5430 | 1.2047 |
| | | NSMRVFG<br>L | 0.6202 | 1.167<br>3 | 0.1322 | 0.6170 | 1.2179 |
| | | LVAIFDAF<br>K | 0.4308 | 0.810<br>9 | 0.1346 | 0.5190 | 0.8570 |
| | | FLLTWILF<br>R | 0.7171 | 1.349<br>7 | 0.4207 | 1.5810 | 1.4919 |

| | | | | | | | |
|---|---|---|---|---|---|---|---|
| | | GLQGYLTEK | 0.5733 | 1.0790 | 0.8523 | 0.1680 | 1.2152 |
| | | KVYQSTYFL | 0.3786 | 0.7126 | 0.9548 | 1.2200 | 0.9168 |
| | | YQSTYFLSY | 0.2782 | 0.5235 | 0.9740 | 3.0230 | 0.820 |
| | | TIGHIFPLY | 0.5432 | 1.0224 | 0.9419 | 3.0230 | 1.3148 |
| | | IFPLYFKFK | 0.3348 | 0.6301 | 0.6617 | 0.6170 | 0.7602 |
| | | WIMITLITK | 0.3304 | 0.6218 | 0.7680 | 0.6400 | 0.7690 |
| | | IMITLITKY | 0.5556 | 1.0457 | 0.9254 | 3.0820 | 1.3387 |
| | | IILIPWLDY | 0.4035 | 0.7593 | 0.9630 | 3.1410 | 1.0608 |
| | | YFFNSDPLK | 0.3384 | 0.6368 | 0.6378 | 0.7720 | 0.7711 |
| | | IILFFCLWY | 0.5161 | 0.9713 | 0.2810 | 3.0420 | 1.1656 |
| | | NIIRILHGK | 0.3385 | 0.6372 | 0.5807 | 0.8530 | 0.7669 |
| | | RILHGKESK | 0.5574 | 1.0491 | 0.4658 | 0.8640 | 1.1622 |
| | Q9ZB71_Y3 | FLFVPLLIY | 0.4618 | 0.8692 | 0.8914 | 2.9050 | 1.1482 |
| | | AFFLFAFTK | 0.5489 | 1.0331 | 0.9039 | 0.9360 | 1.2155 |
| | | MLSFFLLNK | 0.7774 | 1.4631 | 0.6930 | 0.5750 | 1.5958 |
| | | KMTNFFLIR | 0.5161 | 0.5931 | 1.1163 | 0.6506 | 1.8180 |
| | | TNFFLIRSK | 0.3753 | 0.7063 | 0.1975 | 0.4390 | 0.7578 |
| | | IIIAENLLK | 0.6365 | 1.1980 | 0.4444 | 0.8390 | 1.3066 |
| | | NLLKTGKSK | 0.4275 | 0.8046 | 0.5825 | 0.3720 | 0.9106 |
| | | KSKTKNRQK | 0.5212 | 0.9810 | 0.5240 | 0.5800 | 1.088 |
| | | IVFKIKKKK | 0.6353 | 1.1957 | 0.8213 | 0.5710 | 1.3474 |
| **A24 supertype** | | | | | | | |
| | sp_P47377_Y1pep | QYSALIPLF | 0.7529 | 1.6031 | 0.2163 | 2.8440 | 1.7778 |
| | | YSALIPLFI | 0.4414 | 0.9400 | 0.0755 | 0.5230 | 0.9775 |
| | | ILLISLVLF | 0.3194 | 0.6802 | 0.9299 | 2.4900 | 0.9442 |
| | | LISLVLFCF | 0.2848 | 0.6064 | 0.9359 | 2.7110 | 0.8823 |
| | | NQIVKILFF | 0.3053 | 0.6502 | 0.4183 | 2.5490 | 0.840 |
| | | FFAYCIDFL | 0.4469 | 0.9517 | 0.0680 | 1.0920 | 1.0165 |

| | | | | | | | |
|---|---|---|---|---|---|---|---|
| | | AYCIDFLAL | 0.4822 | 1.0267 | 0.5117 | 1.2670 | 1.1668 |
| | | ILAVMLLTF | 0.3839 | 0.8174 | 0.7740 | 2.6420 | 1.0656 |
| | sp_P58061_SE | MHPIQIVMF | 0.2828 | 0.6022 | 0.5042 | 2.5720 | 0.8064 |
| | | MFIMAVICL | 0.4154 | 0.8846 | 0.3396 | 1.2850 | 0.9998 |
| | | GFVKILQII | 0.3355 | 0.7144 | 0.0748 | 0.7330 | 0.7623 |
| | | IMFILVVLF | 0.4650 | 0.9902 | 0.7249 | 2.8000 | 1.2389 |
| | | MFILVVLFL | 0.5957 | 1.2685 | 0.5624 | 1.2470 | 1.4152 |
| | | FILVVLFLI | 0.3428 | 0.7298 | 0.2226 | 0.6220 | 0.7943 |
| | | LFLILGLIF | 0.5535 | 1.1786 | 0.7416 | 2.7710 | 1.4284 |

Table S3. Galaxy refine structure data

| **Model** | **GDT-HA** | **RMSD** | **MolProbity** | **Clash score** | **Poor Rotamers** | **Rama favoured** |
|---|---|---|---|---|---|---|
| MODEL 1 | 0.9119 | 0.507 | 2.375 | 18.0 | 0.3 | 87.0 |
| MODEL 2 | 0.9215 | 0.487 | 2.413 | 20.3 | 0.9 | 87.5 |
| MODEL 3 | 0.9107 | 0.516 | 2.404 | 19.9 | 0.6 | 87.5 |
| MODEL 4 | 0.9119 | 0.515 | 2.426 | 21.5 | 0.3 | 88.0 |
| MODEL 5 | 0.9089 | 0.515 | 2.417 | 21.1 | 0.3 | 88.0 |
| MODEL 1 | 0.9119 | 0.507 | 2.375 | 18.0 | 0.3 | 87.0 |

| Sec.Cons. : | | | | | |
|---|---|---|---|---|---|
| Alpha helix | (Hh) | : | 247 | is | 59.38% |
| $3_{10}$ helix | (Gg) | : | 0 | is | 0.00% |
| Pi helix | (Ii) | : | 0 | is | 0.00% |
| Beta bridge | (Bb) | : | 0 | is | 0.00% |
| Extended strand | (Ee) | : | 28 | is | 6.73% |
| Beta turn | (Tt) | : | 0 | is | 0.00% |
| Bend region | (Ss) | : | 0 | is | 0.00% |
| Random coil | (Cc) | : | 136 | is | 32.69% |

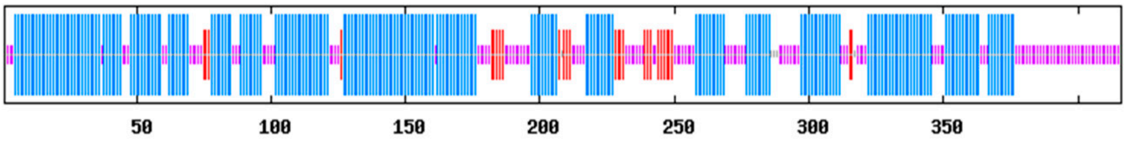


**Figure S1.** The graphical representation of the secondary structure configuration of the multi-epitope vaccine as predicted by the NPS@ server

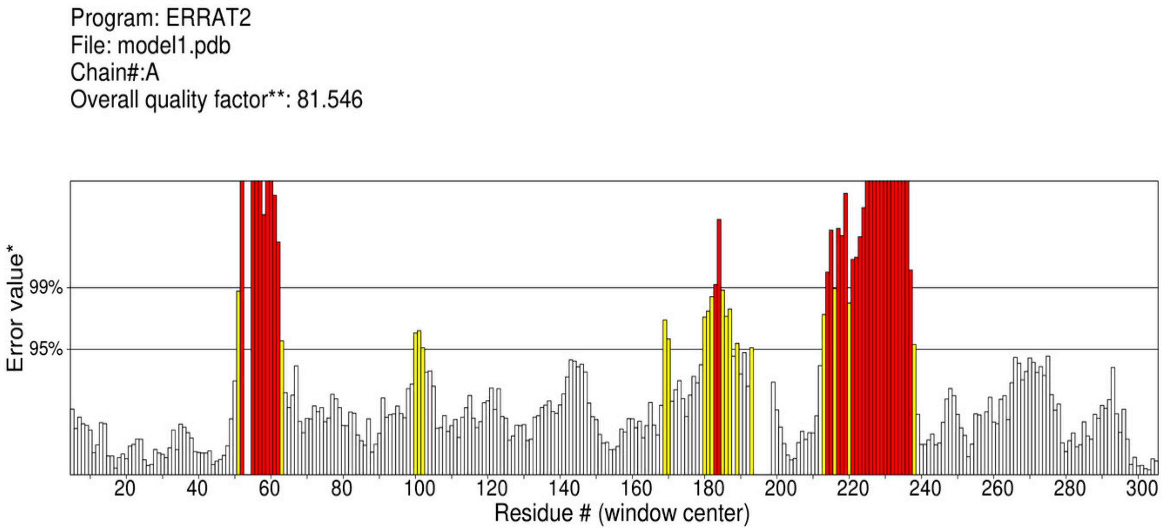


**Figure S2.** Overall quality factor as projected by ERRAT

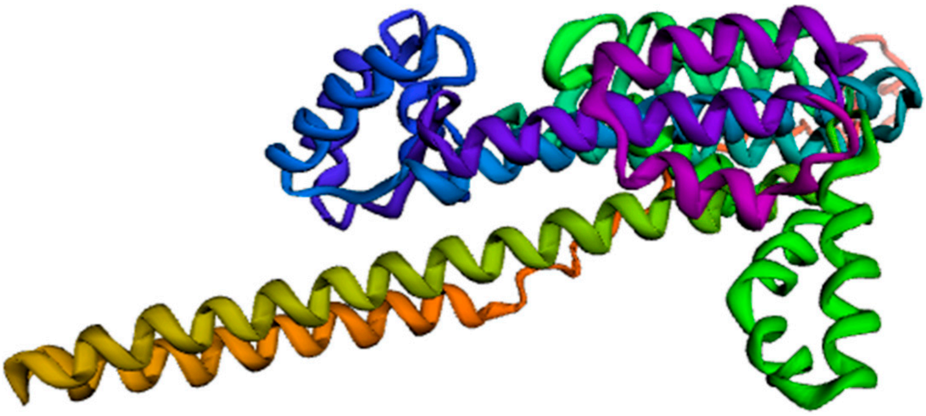

**Figure S3.** 3D model of vaccine construct as generated by Robetta
